# The hippo signaling pathway: implications for heart regeneration and disease

**DOI:** 10.1186/s40169-014-0027-0

**Published:** 2014-09-16

**Authors:** Dominic P Del Re

**Affiliations:** Cardiovascular Research Institute and Department of Cell Biology and Molecular Medicine, New Jersey Medical School, Rutgers Newark, 07103 NJ USA

**Keywords:** Hippo signaling pathway, Heart disease, Myocardial regeneration

## Abstract

Control of cell number and organ size is critical for appropriate development and tissue homeostasis. Studies in both *Drosophila* and mammals have established the Hippo signaling pathway as an important modulator of organ size and tumorigenesis. Upon activation, this kinase cascade modulates gene expression through the phosphorylation and inhibition of transcription co-activators that are involved in cell proliferation, differentiation, growth and apoptosis. Hippo signaling serves to limit organ size and suppress malignancies, and has been implicated in tissue regeneration following injury. These outcomes highlight the important role that Hippo signaling plays in regulating both physiologic and pathologic processes. In this review, an overview of the signaling pathway will be discussed as well as recent work that has investigated its role in cardiac development, regeneration and disease.

**Electronic supplementary material:**

The online version of this article (doi:10.1186/s40169-014-0027-0) contains supplementary material, which is available to authorized users.

## Introduction

Myocardial infarction (MI), or insufficient blood flow to the heart muscle, promotes thedeath and loss of cardiomyocytes resulting in heart damage and impaired cardiacfunction. While patient survival following MI has improved, the prognosis is typicallypoor and can eventually progress to heart failure, a leading cause of morbidity andmortality [1]. Because mature cardiomyocytes have a limited capacity to re-enter the cellcycle and proliferate [2],[3], the ability of the adult heart to regenerate is similarlyrestricted and cannot adequately replace lost cardiomyocytes. The Hippo signalingpathway is evolutionarily conserved from flies to mammals and has emerged as animportant regulator of both cell survival and proliferation [4],[5]. Importantly, this cascadealso appears critical for proper mammalian heart development and the post-natal responseto cardiac stress and injury[6-8]. It is therefore plausible to hypothesize that Hipposignaling could be targeted to promote heart regeneration after MI and heart injury. Thisreview will provide an overview of the Hippo pathway and examine its role in cardiacdevelopment, disease and regeneration.

## Review

### Hippo signaling in *Drosophila*

Determining how multicellular organisms attain and maintain appropriate organ size has plagued researchers and remains a major quest. Studies employing genetic mosaic screens in *Drosophila melanogaster* shed new light on this question and identified the core components of what is now known as the Hippo signaling pathway, appropriately named after Hippo kinase. Mutant flies genetically deficient for a functional Hippo (Hpo) [1]-[5], Salvador (Sav) [6],[7] or Warts (Wts) [8],[9] exhibited similar phenotypes consisting of robust tissue overgrowth, enhanced cell proliferation and suppressed apoptosis. These shared phenotypes suggested the involvement of each in a common signaling cascade that regulated organ size, which was subsequently demonstrated through additional genetic and protein interaction studies. Integral work revealed that Hippo (a serine/threonine kinase) interacts with Salvador (a WW-repeat protein and scaffold) and promotes the phosphorylation of both Salvador and Warts kinase [1]. A fourth core component, Mats (Mob as tumor suppressor) is also phosphorylated by Hippo and was found to play an important role in this cascade by interacting with Warts, which may further facilitate Warts activation [10],[11]. These seminal studies demonstrated how the association and phosphorylation between core components promoted Hippo pathway activation.

In 2005, work from Pan and colleagues provided the critical link between the Hippo cascade and gene expression. Using a yeast two-hybrid approach with Warts as bait, they were able to identify the transcription co-activator Yorkie (Yki) as a downstream target of Hippo signaling [12]. Subsequent work demonstrated that Yorkie is a direct substrate of Warts and that Yorkie promotes cell proliferation and growth. Importantly, inactivation of Yorkie was sufficient to prevent the overgrowth phenotype observed in Hippo pathway mutants, indicating that Yorkie is the major downstream effector of the pathway [12],[13]. Yorkie itself lacks the ability to bind DNA directly and therefore influences gene expression through its interaction with the transcription factor Scalloped (Sd) [14]-[17]. Scalloped was demonstrated to mediate Yorkie-triggered overgrowth; however, overexpression of Scalloped alone did not elicit overgrowth, suggesting that Yorkie regulation is the critical factor for determining gene expression downstream of Hippo signaling [14].

## Hippo signaling in mammals

There is high conservation of Hippo signaling components between *Drosophila* and mammals. Similar to the fly, mammalian Hippo signaling involves the activation of a conserved kinase cascade that ultimately phosphorylates the transcription co-activator Yap (Yes-associated protein) and its paralog TAZ (transcriptional co-activator with a PDZ-binding motif; both are orthologs of *Drosophila* Yki), thereby regulating downstream gene expression, cell proliferation and organ growth (for excellent reviews, see [18]-[22]). The core components of the mammalian Hippo pathway include the serine/threonine kinases Mst1/2 (mammalian sterile 20-like kinase; ortholog of *Drosophila* Hpo) and Lats1/2 (large tumor suppressor; ortholog of *Drosophila* Wts), as well as the adapter proteins Salvador (Sav1; also referred to as WW45) and Mob1 (Mps one binder; ortholog of *Drosophila* Mats)[23]. Analogous to Hippo signaling in the fly, Mst1/2 associates with and phosphorylates Sav1, which further promotes Mst1/2 activation. Once activated, Mst1/2 directly phosphorylates and activates Lats1/2[24]. Mst1/2 can also phosphorylate Mob1, which may further promote pathway activation through a currently undefined mechanism. Interestingly, Hippo signaling appears to have a high degree of evolutionary conservation from flies to humans as mammalian orthologs exist for each fly counterpart and generally function in a similar manner. However, it should be noted that recent work has demonstrated a clear divergence between select upstream pathway components (e.g., *Drosophila* Fat and Expanded, see below) and may explain why regulation of core Hippo signaling by these orthologs appears inconsistent between flies and mammals [25]. Furthermore, Yorkie/Yap transcriptional differences have been observed between flies and mammalian cell types highlighting differences in Hippo signaling outputs between species [26].

## Regulation of gene expression by the Hippo pathway

Like many other established signal transduction pathways, Hippo signaling regulates a transcriptional program. The identification of Yorkie as a direct substrate of Warts provided the important link between the Hippo core components and gene expression [12],[13],[27]. As mentioned above, Yorkie does not bind DNA and partners with Scalloped to mediate gene expression, cell proliferation and survival [15]-[17]. Several gene targets of Hippo signaling have been identified in *Drosophila,* including *cyclin E*, *diap1*, and the microRNA *bantam*, which may modulate cell proliferation and survival [1],[12],[14],[28],[29]. Similarly, the Yorkie orthologs Yap/TAZ cannot bind DNA directly and modulate gene expression through transcription factor interaction. Perhaps the most established are the TEAD1-4 (TEA domain family member) transcription factors (orthologs of *Drosophila* Sd), which have been shown to mediate many of the cellular effects of activated Yap/TAZ [17],[30]-[44]. In addition to transcriptional partnering with TEADs, Yap/TAZ have the ability to co-activate or co-repress other known transcription factors including p73 [45], ErbB-4 [46], Runx [47],[48], FoxO1 [49], Tbx5 [50] and Smads [51]-[53]; however their respective roles in Hippo signaling are not clear.

Yap has been the focus of intense research since it was discovered to be the link between Hippo signaling and gene expression. Yap contains five potential Lats phosphorylation sites (HXRXXS) and mutation of one of these sites, a serine to alanine substitution at residue 127, was shown to attenuate Yap phosphorylation by Lats2 and modulate its subcellular distribution from cytosolic to nuclear prevalence [13],[27],[54],[55]. Serine 127 phosphorylation promotes 14-3-3 binding and Yap cytosolic retention and led to a decrease in Yap-TEAD interaction, although this modification did not directly alter Yap-TEAD binding [13]. Additional phosphorylation of Yap at serine 381 serves as a phosphodegron and promotes its proteosomal degradation [56]. Interestingly, recent work has shown that PKA-mediated phosphorylation of Lats2 promotes its kinase activity toward Yap, but is selective for serine 381, thereby enhancing Yap degradation [57]. These findings suggest that distinct signaling may bias Lats-mediated phosphorylation and subsequent regulation of Yap.

Yap activity is also regulated by multiple upstream signaling pathways that are independent of Hippo signaling. Yap was originally discovered as a WW-domain-containing protein that interacted with Yes, a member of the Src family of tyrosine kinases [58]. Subsequent work has shown that Yap is phosphorylated by Yes/Src and that this is required to enable Yap modulation of Runx2-mediated transcription [48]. Yap/TAZ also display extensive crosstalk with Wnt/β-catenin signaling. Activation of Hippo signaling and increased phosphorylation of Yap/TAZ has been shown to promote their interaction with β-catenin, increasing cytosolic retention of β-catenin, and thereby negatively regulating Wnt/β-catenin signaling [59]. Additionally, inhibition of Hippo and activation of Yap caused increased β-catenin-mediated gene expression in the heart, and β-catenin was found to be required for the activated Yap phenotype [60]. Similar findings were observed using a constitutively active Yap mutant – namely that active Yap promotes β-catenin target gene expression both directly and indirectly (through activation of IGF-1 signaling) and that β-catenin is a critical mediator of Yap function [61]. These findings suggest that Yap is a positive regulator of the Wnt/β-catenin pathway. Conversely, studies have also demonstrated that Wnt/β-catenin signaling regulates the function of Yap/TAZ. Activation of Wnt causes stabilization of Yap through its target tribbles homolog 2 (TRIB2) [62]. Wnt can also prevent degradation of TAZ by preventing the phosphorylation of β-catenin, which serves as a bridge that links TAZ to its ubiquitin ligase β-TrCP [63]. Recently published work has further clarified the underlying mechanism by demonstrating that Yap/TAZ are integral components of the β-catenin destruction complex and that Wnt activation triggers the nuclear aggregation of both β-catenin and Yap/TAZ [64]. Additional reports have implicated the involvement of JNK in regulating Yap activity either directly through JNK phosphorylation of Yap [65] or indirectly by promoting the interaction of Lats with Ajuba family proteins thereby limiting the Lats-Yap inhibitory interaction [66]. Taken altogether, these studies highlight the crosstalk surrounding Yap/TAZ and suggest they are key nodes of signaling transduction.

## Upstream modulators of Hippo signaling in *Drosophila*

As the importance of Hippo signaling becomes increasingly apparent, significant efforts have been made to clarify the proximal signaling responsible for pathway modulation. In *Drosophila*, a number of potential upstream components have been identified as important pathway regulators. Genetic screens identified the FERM domain family members Merlin (Mer) and Expanded (Ex). Mutations in either of these genes led to an attenuation of Hippo signaling in flies [67]. Interestingly, Expanded was shown to negatively regulate Yorkie directly through a protein-protein interaction, which led to cytosolic sequestration and increased Yorkie degradation [68],[69]. The mechanism responsible for linking Merlin to the core Hippo kinase cassette remained elusive until elegant work from Pan and colleagues revealed a potential mechanism. Their study found that membrane-associated Merlin binds and recruits Warts to the plasma membrane. Here, Warts is phosphorylated by an independent Hippo/Salvador complex, thereby promoting pathway activation. Of note, this work was unique in demonstrating spatial compartmentalization of this signaling pathway [24]. Additional studies have implicated Kibra, a protein known to interact with both Merlin and Expanded, as a potential regulator of Hippo signaling. Indeed, loss of Kibra function appears to phenocopy *mer* and *ex* mutants, while Kibra overexpression caused increased phosphorylation of Hippo, Warts and Yorkie [70]-[72]. Fat, an atypical protocadherin, has also been identified as an upstream regulator, and possible membrane receptor, of Hippo signaling in the fly [73]-[77]. Fat mutants (loss-of-function) displayed overgrowth phenotypes similar to those of Hippo pathway loss-of-function mutants, as well as dysregulation of Hippo phosphorylation and Yorkie target gene expression [73]-[75]. Furthermore, Fat modulated protein levels and proper localization of Expanded, identifying a potential mechanism that links Fat to Hippo. Daschous, another transmembrane cadherin, also regulates Hippo signaling through its interaction with Fat and Zyxin, ultimately modulating Warts protein levels [78]. Crumbs, a known cell polarity factor in *Drosophila*, has also been implicated in Hippo signaling [53],[79],[80]. Loss of Crumbs caused mislocalization of Expanded and inhibition of Hippo activation, leading to hyperactivation of Yorkie and increased tissue growth [81],[82]. It has also been demonstrated that Hippo is phosphorylated in its activation loop by Tao-1, another member of the sterile 20-like kinase family. Phosphorylation of Hippo by Tao-1 promoted the activation of core Hippo components and led to restrained Yorkie function [83],[84]. Recent work from Wehr et al. employed a genome-wide RNAi screen in *Drosophila* and identified the salt-inducible kinases, Sik2 and Sik3, as negative regulators of Hippo signaling [85]. The Siks were found to disrupt Hippo/Warts complex formation through phosphorylation of Salvador, leading to increased Yap activation. As these kinases may be sensitive to nutrient sensing, they could provide a link between extracellular cues and growth through the Hippo pathway. Yet another kinase, homeodomain-interacting protein kinase 2 (HIPK2) has been demonstrated to regulate Hippo pathway activity. However, HIPK2 does not appear to modulate upstream Hippo members, but instead directly phosphorylates and activates Yorkie to promote increased tissue growth [86],[87]. Given the intense interest in Hippo signaling, this burgeoning list of pathway components continues to expand. In this regard, for a more in-depth discussion of additional regulators of the Hippo pathway, the reader is referred to these excellent reviews [21],[88]-[92].

## Upstream modulators of mammalian Hippo signaling

Although many *Drosophila* Hippo pathway components have mammalian orthologs, and although many of these have been demonstrated to have similar functions to their fly counterparts, great care must be exercised when investigating Hippo signaling in mammalian systems as it most likely will prove to be more complex and nuanced. In fact, clear differences between flies and mammals with regard to Hippo signaling have been demonstrated [25],[26],[93]. Nonetheless, substantial effort has been directed toward elucidating the proximal regulators of mammalian Hippo signaling in recent years and significant progress has been made on this front. RASSF1A (Ras association domain family 1A) is a tumor suppressor and scaffold protein that has been shown to interact with Mst1/2 and Sav1 to promote activation of Hippo signaling [94],[95]. RASSF1A causes increased Mst1/2 autophosphorylation, at least in part through suppression of Mst1/2 dephosphorylation by PP2A [96], thereby promoting Mst1/2 activation and subsequent apoptosis [97],[98]. It should be noted that the *Drosophila* ortholog of RASSF1A (dRASSF) was demonstrated to prevent Hippo activation [93], a reminder of the potential differences between flies and mammals. Studies involving the tumor suppressor NF2 (Neurofibromin 2; ortholog of Merlin) have demonstrated its role in regulating mammalian Hippo signaling. Targeted deletion of the *Nf2* gene in the mouse liver (albumin-Cre driver specific for hepatocytes and biliary cells) led to increased Yap activation, increased cell proliferation, increased liver size and eventual hepatocellular carcinoma (HCC)[99],[100]. Interestingly, this NF2 KO phenotype was completely prevented in mice that were also haplo-deficient for *Yap*, indicating that augmented Yap activity is responsible for this pathology [99]. On the other hand, treatment with an EGFR (epidermal growth factor receptor) inhibitor also prevented the overgrowth caused by *Nf2* deletion, suggesting that multiple pathways downstream of NF2 may be altered and contribute to HCC [100]. Loss of NF2 function in the dorsal telencephalon of the murine brain also elicited increased Yap/TAZ activation and increased cell proliferation/expansion leading to developmental defects [101]. While these results implicate a role for NF2 in regulating Hippo signaling in two distinct tissues, additional work has demonstrated a similar anti-proliferative but non-Hippo-related effect. Specifically, NF2 was found to translocate to the nucleus where it inhibited the E3 ligase DCAF1 to suppress cell proliferation independent of Hippo signaling [102]. Taken together, RASSF1A and NF2 appear to be positive modulators that promote mammalian Hippo signaling in multiple tissue types.

The ability to sense and transduce extracellular signals and/or cues from the surrounding environment into the cell is a hallmark of traditional signaling pathways. Along these lines, work from Guan and colleagues demonstrated that cell density regulates the activation status of Hippo signaling [13]. Cell-to-cell contact, long known to cause inhibition of proliferation, was linked to the inactivation of Yap. Importantly, this work demonstrated the involvement of the Hippo pathway in regulating cell proliferation in response to the cell density signal. Further studies revealed that β-catenin is an important mediator of the cell density signal and a direct regulator of Yap phosphorylation and degradation in epithelial stem cells [39]. In a similar vein, Kim et al. reported that E-cadherin regulates Yap exclusion from the nucleus in a cell-density-dependent context [103]. Yap inhibition and cytosolic retention caused by cell-cell contact was mediated by E-cadherin, and forced Yap expression was able to overcome the anti-proliferative effect of E-cadherin. For excellent reviews regarding Hippo regulation by cell-cell contact and cell polarity see [91],[104].

**Figure 1:**
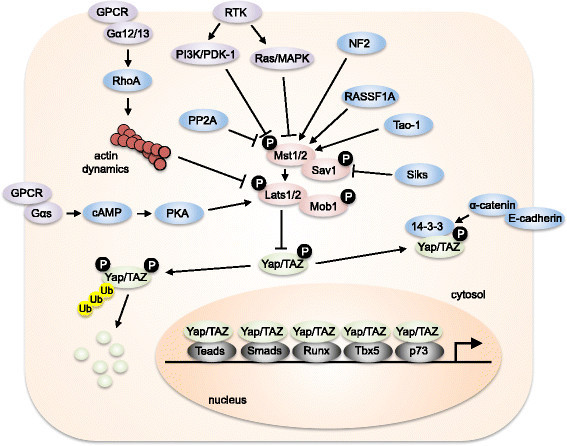
**A schematic representation of mammalian Hippo signaling.** The core components of the Hippo pathway in mammals consist of Mst1/2, Lats1/2, Salvador (Sav1) and Mob1. Active Lats1/2 phosphorylates and inhibits Yap/TAZ. Yap phosphorylation on Serine 127 results in 14-3-3 binding and cytosolic retention (right side) while Serine 381 phosphorylation leads to proteosomal degradation (left side). Nuclear Yap/TAZ regulate gene expression resulting in proliferation, growth and survival. Yap/TAZ association with several identified transcription factors is shown. Arrows and blunt lines indicate activation and inhibition, respectively.

In a landmark study, the Guan group was the first to demonstrate that extracellular receptors, in this case GPCRs (G-protein-coupled receptors), could modulate Hippo signaling [105]. Ligands that preferentially couple to Gα12/13 (e.g., LPA, S1P), and to a lesser extent Gαq, were shown to inhibit Hippo signaling and promote Yap activation. Conversely, ligands that preferentially activate Gαs (e.g., epinephrine) stimulated Hippo signaling and inhibited Yap activity. Follow-up work described similar findings for PARs (protease-activated receptors), a subset of GPCRs that is stimulated by the endogenous ligand thrombin [106]. Further independent work confirmed that the extracellular diffusible signals LPA and S1P are indeed potent activators of Yap [107]. Interestingly, these ligands appear to act through the actin cytoskeleton and the small G-protein RhoA, as the signaling cascade is sensitive to actin disruption and inhibition of RhoA, and regulate the function of Lats kinases to control Yap activity [105],[107],[108]. Further investigation into the connection between cytoskeletal integrity and Lats identified a role for PKA (cAMP-dependent protein kinase) in the regulation of Hippo signaling. cAMP/PKA were shown to cause Yap/TAZ phosphorylation and inhibition through a process requiring actin polymerization and RhoA [57],[108]. Kim et al. also demonstrated direct phosphorylation and activation of Lats2 by PKA. This modification appeared to bias Lats2 kinase activity toward inhibitory phosphorylation of Yap at serine 381 but not serine 127 [57]. In addition to GPCR-dependent modulation of Hippo signaling, two compelling studies demonstrated a role for receptor tyrosine kinases (RTKs) in regulating this cascade. Fan et al. demonstrated that activation of the EGF receptor in mammary cells caused the activation of Yap [109]. Mechanistically, the authors demonstrated that this was mediated by PI3K and PDK1, which are activated downstream of EGF receptor engagement, but did not require AKT. Instead, PDK1 associates with Salvador to disrupt the core Hippo signaling components, leading to Lats inhibition and Yap activation. A similar finding was reported by Reddy and Irvine [110]. Here, activation of EGFR elicited Ras/MAPK activation and phosphorylation of the Ajuba family protein WTIP (Wilms tumor protein 1-interacting protein). The fly homolog of WTIP, Jub, was also phosphorylated by MAPK, causing it to bind to Salvador and Warts, inhibiting Warts and eliciting Yorkie activation (see Figure 1).

Additional groundbreaking studies from the Piccolo lab have shown that extracellular matrix (ECM) stiffness and mechanotransduction systems can also modulate the activity of Yap/TAZ [111]. This work demonstrated that Yap/TAZ are a critical link between ECM rigidity and altered gene expression, which is known to affect cell growth, differentiation and proliferation. The cell's ability to spread and the amount of mechanical stress to which it is subjected were identified as key regulators of Yap/TAZ nuclear localization and transcriptional activity. The underlying mechanism was found to be mediated by RhoA and actomyosin tension; however, Mst/Lats kinases appeared to be dispensable. Further work using a siRNA screen of F-actin-associated proteins identified and subsequently confirmed Cofilin, CapZ and Gelsolin as important inhibitors of Yap/TAZ in cells experiencing low mechanical stress [112]. Taken altogether, regulation of mammalian Hippo signaling is complex and likely incorporates multiple upstream inputs to determine intensity and duration of signal activation. Furthermore, it is likely that regulation and function of this pathway are cell- and tissue-type-specific and additional investigation is therefore needed.

## Hippo signaling and heart development

The mammalian heart develops from mesodermal tissues, first forming a heart tube, which then loops and gives rise to atrial and ventricular components and an outflow tract. These complex processes require finely orchestrated spatial-temporal signaling events between multiple cell types to give rise to a normal, healthy heart [113]. Tbx5 is a transcription factor and one of several known regulators of embryonic heart development [114]. Importantly, Tbx5 can be co-activated by Yap/TAZ, indicating the possible involvement of Hippo signaling in early stage heart development [50].

Recent studies have begun to further examine the role of Hippo in mammalian cardiogenesis. A mouse model harboring embryonic deletion of *Sav1, Mst1/2 or Lats2* in the heart revealed the importance of these Hippo components for proper organ development [60]. All knockout lines displayed a similar phenotype and showed hyperproliferation of cardiomyocytes with thickened ventricular walls due to an excessive cardiomyocyte number. These mice also died prior to birth. Mechanistically, there was significant upregulation of Wnt target gene expression, which was rescued by crossing the Hippo mutant mice with those deficient for β-catenin. These findings suggest that Hippo restrains cardiomyocyte proliferation and heart size by inhibiting Wnt signaling and point to the importance of Hippo signaling during cardiogenesis. In a complimentary approach, two additional groups utilized genetically altered mice that harbored embryonic deletion of *Yap* in the heart to interrogate the function of endogenous Yap during heart development [61],[115]. Importantly, two different promoters were used to drive cardiomyocyte-specific Cre recombinase expression; however, similar results were observed between the two lines including a decrease in cardiomyocyte proliferation, a decrease in pro-growth and proliferative gene expression (the former via Wnt/β-catenin and the latter via TEAD), and embryonic lethality between E10.5-12.5. Taken together, these findings demonstrate the critical involvement of Hippo signaling during cardiogenesis and suggest that a balance of Yap activity is required for proper heart formation (see Table 1 for overview of Hippo-related mouse models and cardiac phenotypes).

**Table 1:** **Overview of Hippo-related mouse models**

Mouse line	Cardiac phenotype	Yap activity	References
Mst1 Tg (αMHC)	Dilated cardiomyopathy, increased apoptosis and no compensatory hypertrophy. Premature death.	ND	[129]
DN-Mst1 Tg (αMHC)	Normal cardiac function. Protection against I/R and MI.	ND	[129],[130]
Lats2 Tg (αMHC)	Baseline cardiac dysfunction. Reduced heart size.	ND	[135]
DN-Lats2 Tg (αMHC)	Cardiac hypertrophy with normal cardiac function. Protection against I/R.	ND	[135]
Sav1 cKO (Nkx2.5-cre)	Embryonic lethal. Cardiomegaly, thickened walls and increased proliferation.	Increased	[60]
Mst1/2 cKO (Nkx2.5-cre)	Embryonic lethal. Enlarged heart.	ND	[60]
Lats1/2 cKO (Nkx2.5-cre)	Embryonic lethal. Enlarged heart.	ND	[60]
Yap Tg (αMHC)	Enlarged heart with increased proliferation. Increased cardiac regeneration.	Increased	[123]
Yap Tg (αMHC)	Enlarged heart with Increased proliferation.	Increased	[115]
Yap Tg (αMHC; ROSA26^fs-rtTA^; TRE-Yap)	Normal cardiac function. Increased proliferation. Protection against MI with no change in apoptosis.	Increased	[124]
Yap cKO (Nkx2.5-cre)	Embryonic lethal. Reduced heart size, reduced proliferation.	Reduced	[61],[115]
Yap cKO (Tnnt2-cre)	Embryonic lethal. Reduced heart size, reduced proliferation.	Reduced	[115]
Yap cKO (αMHC-cre)	Dilated cardiomyopathy, increased apoptosis, increased fibrosis and premature death. Worsened outcome after MI (het).	Reduced	[122],[123]
Sav1 cKO (αMHC-MCM)	Normal cardiac function. Increased proliferation and heart regeneration. Protected against MI.	Increased	[125]
Lats1/2 cKO (αMHC-MCM)	Normal cardiac function. Increased proliferation and heart regeneration. Protected against MI.	Increased	[125]

A summary of Hippo pathway gain- and loss-of-function mouse models with corresponding cardiac phenotypes and effects on Yap activity. ND = not determined.

## Hippo signaling in cardiac regeneration

Embryonic heart growth occurs mainly through proliferation of cardiomyocytes, whereas shortly after birth these cells experience a dramatic reduction in their ability to divide. Thus, further organ growth is almost exclusively mediated through the enlargement of existing cardiomyocytes. Around this same time, the ability of the mammalian heart to regenerate following injury drastically declines. It should be noted, however, that low-level cardiomyocyte turnover seems to occur throughout adult mammalian life [116],[117] and approaches aimed at exploiting this phenomenon are an active area of cardiovascular research. This is in contrast to the zebrafish, which is able to regenerate up to 20% of its cardiac mass following myocardial resection, even during adulthood [118]. Because the loss of cardiomyocytes in response to injury is a major contributor to impaired function and heart failure, it is fundamental to understand the mechanisms that regulate proliferation and consequently regeneration of adult mammalian cardiomyocytes. Yap has been demonstrated to promote the self-renewal and "stemness" of embryonic stem cells [119], satellite muscle cells [120] and liver cells [121], while suppressing or even reversing their differentiation, indicating that Yap could potentially be targeted to manipulate precursor cell fate and perhaps modify regenerative capacity.

Several studies have demonstrated the ability of Yap to promote proliferation of neonatal rat cardiomyocytes. By using adenoviral overexpression systems, these gain-of-function approaches upregulated active Yap and elicited increases in Ki-67-positive, phosphorylated histone H3-positive, and BrdU-positive cardiomyocytes in culture [61],[115],[122]. Ectopic expression of activated Yap *in vivo* also caused increased cardiomyocyte proliferation, indicating that this observation is not limited or specific to neonatal cells [115],[123],[124]. Similarly, conditional deletion of *Sav1* in the adult mouse heart, which causes increased Yap activation, also triggered increased cardiomyocyte proliferation [125], providing further evidence that Hippo signaling restrains adult cardiomyocyte renewal. Recent work has demonstrated that neonatal mouse hearts have a regenerative capacity similar to zebrafish during the first 7 days after birth (P1-7); however, following P7 this capacity for heart renewal declines dramatically [126]. To determine whether Hippo signaling regulates neonatal heart regeneration, Yap transgenic mice were generated. Increased cardiac Yap expression was able to protect against cardiomyocyte loss, fibrosis and scarring after chronic myocardial infarction (MI) in mice beginning at P7. Conversely, cardiac-specific deletion of *Yap* ablated the neonatal regenerative capacity observed in wild-type mice following MI. Yap CKO mice had larger scar areas while wild-type neonates appeared normal [123], suggesting that Yap is a critical mediator of heart regeneration shortly after birth. Additional studies utilized inducible cardiac Sav1 and Lats1/2 CKO mice to test whether these Hippo pathway components could also influence the regenerative capacity of the mammalian heart [125]. Conditional deletion of *Sav1* or *Lats1/2* in neonatal mice was shown to promote heart repair and extend the regenerative potential following cardiac resection at P8. Furthermore, Hippo deficiency led to increased cardiomyocyte proliferation and myocardial protection in response to MI either at P8 or in 8-week-old adult mice. These Sav1 CKO mice also had improved cardiac function and smaller scars after MI compared to wild-type controls. In sum, these studies indicate that Hippo signaling is an important modulator of cardiomyocyte proliferation and myocardial regeneration in response to injury.

## Hippo signaling in heart disease

Mst1 was first cloned and characterized nearly 20 years ago and was later identified as a Hippo ortholog and a core component of the mammalian Hippo signaling pathway [127]. Mst1 is activated during apoptosis of cancer cell lines [128] and cardiomyocytes [129] and, importantly, contributes to their programmed cell death. Myocardial Mst1 transgene expression driven by the cardiomyocyte-specific αMHC promoter led to a profound cardiac phenotype in mice [129]. Mst1 transgenic (Tg) mice developed severe dilated cardiomyopathy at a young age and died prematurely due to heart failure. These Tg mice had elevated caspase activation and cardiomyocyte apoptosis, which contributed to ventricular wall thinning and a decline in cardiac function. On the other hand, using the same approach to express a kinase-inactive (K59R) DN-Mst1 in the heart inhibited endogenous Mst1 activity and afforded cardioprotection [129]. During ischemia and reperfusion (I/R), which simulates acute coronary occlusion and subsequent restoration of blood flow, Mst1 is activated and contributes to cardiomyocyte apoptosis. Indeed, DN-Mst1 Tg mice showed significantly fewer TUNEL-positive cardiomyocytes and significantly reduced scar formation following I/R [129]. Wild-type non-transgenic (NTg) and DN-Mst1 Tg mice were also subjected to chronic MI. DN-Mst1 Tg mice had less apoptosis, reduced fibrosis, attenuation of left ventricle dilation and improved cardiac function compared to controls [130]. Autophagy, an established adaptive mechanism that can protect the heart during stress [131], is also regulated by Mst1 in the adult mouse heart. Recent work demonstrated that Mst1-mediated phosphorylation of Beclin-1 promotes the association of Beclin-1 with Bcl-2 and thereby inhibits cardiomyocyte autophagy [132]. This inhibitory mechanism translates to increased myocardial injury in response to prolonged ischemia. Another recently identified target of Mst1 is the pro-survival Bcl-2 family member Bcl-xL. During I/R, Mst1 was found to translocate to mitochondria and phosphorylate Bcl-xL, causing its dissociation from Bax and leading to Bax activation and cardiomyocyte apoptosis [133]. These findings provide novel mechanisms to explain how Mst1 elicits detrimental outcomes in the heart. Work in pancreatic beta cells has also demonstrated a deleterious role of Mst1 through the promotion of beta cell apoptosis and dysfunction that could contribute to insulin resistance [134]. Together these findings suggest that Mst1 could be a valid therapeutic target not only for the treatment of heart disease, but diabetes as well.

Similar to Mst1, increased expression of Lats2 promotes, while expression of DN-Lats2 attenuates, apoptosis of cultured cardiomyocytes [135]. Interestingly, Lats2 Tg mice had increased myocardial fibrosis and depressed cardiac function, while no significant difference in cardiomyocyte apoptosis was observed under basal conditions. Transgenic expression of DN-Lats2 was used to inhibit endogenous Lats2 in the mouse heart. In response to pressure overload stress (an experimental model of hypertension), DN-Lats2 Tg mice were protected against cardiomyocyte apoptosis and showed an enhanced hypertrophic response, i.e., individual cardiomyocyte enlargement leading to greater overall heart mass [135]. DN-Lats2 Tg mice were also protected against I/R injury and had significantly lower levels of cardiomyocyte apoptosis and scar formation after I/R, which was mediated through increased Yap activation [49]. Taken together, these findings implicate Mst1 and Lats2 as critical mediators of myocardial injury and dysfunction following both acute and prolonged stress.

RASSF1A associates with Mst1/2 and promotes mammalian Hippo signaling [94]-[98]. In the heart, two separate groups utilizing independently generated RASSF1A KO mouse lines reported very similar observations [136],[137]. RASSF1A KO mice had no overt cardiac phenotype at baseline; however, in response to pressure overload, cardiomyocyte hypertrophy was augmented compared to wild-type mice. No apparent differences in cardiac function were observed between wild-type and RASSF1A KO mice. Interestingly, cardiac fibrosis was enhanced in RASSF1A KO mice following pressure overload [136],[137]. Further comparison between systemic RASSF1A KO mice and cardiac-specific RASSF1A CKO mice revealed a paracrine mechanism involving TNF-α secretion by cardiac fibroblasts that mediated the enhanced growth and fibrosis observed in RASSF1A KO hearts [137]. Importantly, RASSF1A CKO mice had better cardiac function after pressure overload compared to controls, indicating that RASSF1A function is most likely cell-type-dependent. RASSF1A was also shown to promote Mst1 activation both in cultured cardiomyocytes and the mouse heart, which mediated its pro-apoptotic effect [137]. Therefore, it appears that RASSF1A is an important upstream regulator of Mst1 in the mammalian heart, but the overall impact of RASSF1A on the myocardium is a balance between cell-type-specific signaling and interactions.

Yap is an oncogene that can promote tumorigenesis [39],[138]-[144], yet its function in the adult heart has only recently been investigated. Systemic deletion of *Yap* results in embryonic lethality at day E8.5 [145],[146]. Similarly, disruption of *Tead1* also causes embryonic death around E11-12 [138]. Interestingly, *Tead1*-/- embryos showed severe cardiac abnormalities including enlarged pericardial cavities, thin ventricular walls and reduced number of trabeculae [145]. These findings suggest that Yap and TEAD1 may have redundant functions during early embryonic development, one of which may be the regulation of heart formation in mice. To avoid embryonic lethality and allow for the study of Yap function in later stages of development, tissue-specific Yap deletion has been attained and employed [99]. Myocardial postnatal deletion of *Yap* caused a rapid and severe cardiomyopathy with premature death by 10-12 weeks of age [122],[123]. Yap CKO mice had robust increases in cardiomyocyte apoptosis and cardiac fibrosis that were associated with decreased cardiac function, indicating a critical role for Yap in maintaining adult heart homeostasis [122]. TAZ CKO mice have also been generated [123]. Interestingly, postnatal cardiac TAZ deletion did not result in an obvious phenotype and did not appear to alter lifespan. Similarly, hemizygous myocardial *Yap* deletion (Yap+/-) did not elicit a baseline cardiac phenotype compared to control mice [122]. However, following chronic MI stress, during which Yap is activated at the site of injury, Yap+/- mice showed increased cardiomyocyte apoptosis and fibrosis. Furthermore, Yap+/- hearts had significantly fewer proliferating cardiomyocytes, attenuated cardiomyocyte compensatory hypertrophy, and worsened heart function compared to control mice after MI [122]. Conversely, transgenic expression of activated Yap in mouse hearts, either prior to or immediately after MI, protected against MI-induced injury [123],[124]. Increased Yap expression in adult mouse hearts caused an increase in cardiomyocyte proliferation and significantly reduced scar formation [123],[124]. These findings are comparable to those observed in Sav1 CKO hearts after MI, a condition that upregulates Yap activity [125]. Taken altogether, these results indicate that Yap is required for adult heart homeostasis and is strongly cardioprotective, likely through its ability to promote both cardiomyocyte survival and proliferation in response to injury.

## Exploiting Hippo signaling for heart regeneration

Because Yap activation has been shown to 1) regulate fetal heart growth [60],[61],[115], 2) promote cardiomyocyte proliferation in both neonatal and adult cardiomyocytes *in vivo*[115],[122]-[124] and 3) extend the regenerative capacity of the neonatal mouse heart [123],[125], Hippo signaling has become an intriguing potential target of manipulation for heart regeneration. However, sizable challenges exist. Stimulating adult cardiomyocytes to re-enter the cell cycle and proliferate is a major barrier. Even a signal as potent as activated Yap expression cannot drive a subpopulation of adult cardiomyocytes toward division at a rate that is likely to have much regenerative impact. Indeed, Yap-mediated success in neonatal cardiomyocytes is much greater, indicating that fundamental differences emerge with age. Similarly, stimulating Yap activity through Hippo loss of function had comparable effects on adult cardiomyocyte proliferation, raising the question of whether this approach will be viable and how it might be enhanced. Another concern is the potential for tumorigenesis as a result of increased Yap activation since its oncogenic potential has been demonstrated in proliferative tissues. Although the use of stem cells and cardiac progenitor cells for the treatment of MI in patients has been controversial, this remains another potential route of exploration for Hippo manipulation. Yap has been shown to influence stem/progenitor cell potency and differentiation, yet its function in adult cardiac progenitors is not known. It is possible that engineering cardiac progenitors through modulation of Hippo signaling could afford a more robust progenitor population and increased therapeutic benefit. Additionally, the level of Yap expression/activation in adult cardiac progenitor cells is not known and it would be interesting to determine whether Yap could serve as a marker of cardiac progenitors, similar to c-kit, Islet-1, Sca-1 etc., that proves even more efficacious and overcomes the limitations of those populations being isolated and used currently [147].

## Conclusions

The Hippo signaling pathway has risen to prominence and is regarded as an important transduction mechanism that regulates cell growth, proliferation and survival. It has important implications for physiology and pathology that are the focus of intense ongoing research. Studies to elucidate the importance of Hippo signaling in cardiovascular biology and disease continue to shed light and reveal further complexity of this cascade in the mammalian heart. Future work may focus on the following points of outstanding interest: 1) the underlying mechanism explaining how Yap promotes cardiomyocyte proliferation; 2) upstream regulation of Hippo and how various stresses modulate the pathway; 3) possible crosstalk between additional established signaling networks that impact the heart; and 4) targeting Hippo components using small molecules to influence signaling and outcomes.

## Authors’ original submitted files for images

Below are the links to the authors’ original submitted files for images. Authors’ original file for figure 1

## Abbreviations

BrdU: Bromodeoxyuridine
cAMP: Cyclic adenosine monophosphate
ECM: Extracellular matrix
EGFR: Epidermal growth factor receptor
Ex: Expanded
GPCR: G-protein coupled receptor
HCC: Hepatocellular carcinoma
HIPK2: Homeodomain interacting protein kinase
Hpo: Hippo
I/R: Ischemia/reperfusion
Lats: Large tumor suppressor
LPA: Lysophosphatidic acid
MAPK: Mitogen-activated protein kinase
Mats: Mob as tumor suppressor
Mer: Merlin
MI: Myocardial infarct
Mob: Mps one binder
Mst: Nammalian sterile 20-like kinase
NTg: Non-transgenic
PAR: Protease activated receptor
PKA: cAMP-dependent protein kinase
RASSF1A: Ras association domain family
RTK: Receptor tyrosine kinase
S1P: Sphingosine-1-phosphate
Sav: Salvador
Sd: Scalloped
TAZ: Transcriptional co-activator with a PDZ-binding motif
TEAD: TEA domain family member
Tg: Transgenic
TUNEL: Terminal deoxynucleotidyl transferase dUTP nick end labeling
WTIP: Wilms tumor protein 1-interacting protein
Wts: Warts
Yap: Yes-associated protein
Yki: Yorkie
