# Erratum to: The hippo signaling pathway: implications for heart regeneration and disease

**DOI:** 10.1186/s40169-015-0053-6

**Published:** 2015-03-10

**Authors:** Dominic P Del Re

**Affiliations:** New Jersey Medical School, Cardiovascular Research Institute and Department of Cell Biology and Molecular Medicine, Rutgers Newark, 07103 NJ USA

After publication of this review [1] it emerged that the references in the Introduction section were incorrect. Several references were omitted and other references were numbered incorrectly. These errors were introduced during the final production process by the publisher and SpringerOpen apologises for any inconvenience caused.

The relevant Introduction and references have been replaced with:

Myocardial infarction (MI), or insufficient blood flow to the heart muscle, promotes the death and loss of cardiomyocytes resulting in heart damage and impaired cardiac function. While patient survival following MI has improved, the prognosis is typically poor and can eventually progress to heart failure, a leading cause of morbidity and mortality (one). Because mature cardiomyocytes have a limited capacity to re-enter the cell cycle and proliferate [116, 117], the ability of the adult heart to regenerate is similarly restricted and cannot adequately replace lost cardiomyocytes. The Hippo signaling pathway is evolutionarily conserved from flies to mammals and has emerged as an important regulator of both cell survival and proliferation (four) [20]. Importantly, this cascade also appears critical for proper mammalian heart development and the post-natal response to cardiac stress and injury [60, 123, 124]. It is therefore plausible to hypothesize that Hippo signaling could be targeted to promote heart regeneration after MI and heart injury. This review will provide an overview of the Hippo pathway and examine its role in cardiac development, disease and regeneration.

The omitted references can be found below:

Reference one

Go, A. S., Mozaffarian, D., Roger, V. L., Benjamin, E. J., Berry, J. D., Blaha, M. J., Dai, S., Ford, E. S., Fox, C. S., Franco, S., Fullerton, H. J., Gillespie, C., Hailpern, S. M., Heit, J. A., Howard, V. J., Huffman, M. D., Judd, S. E., Kissela, B. M., Kittner, S. J., Lackland, D. T., Lichtman, J. H., Lisabeth, L. D., Mackey, R. H., Magid, D. J., Marcus, G. M., Marelli, A., Matchar, D. B., McGuire, D. K., Mohler, E. R., 3rd, Moy, C. S., Mussolino, M. E., Neumar, R. W., Nichol, G., Pandey, D. K., Paynter, N. P., Reeves, M. J., Sorlie, P. D., Stein, J., Towfighi, A., Turan, T. N., Virani, S. S., Wong, N. D., Woo, D., Turner, M. B., American Heart Association Statistics, C. and Stroke Statistics, S. (2014) Heart disease and stroke statistics--2014 update: a report from the American Heart Association. Circulation. **129**, e28-e292

Reference four

Staley, B. K. and Irvine, K. D. (2012) Hippo signaling in Drosophila: recent advances and insights. Developmental dynamics : an official publication of the American Association of Anatomists. **241**, 3–15
