# Supplementary figures and images for: The hippo signaling pathway: implications for heart regeneration and disease
Source: Clin Transl Med. 2014 Sep 16;3:27. doi: 10.1186/s40169-014-0027-0 (PMC4884045; doi:10.1186/s40169-014-0027-0)

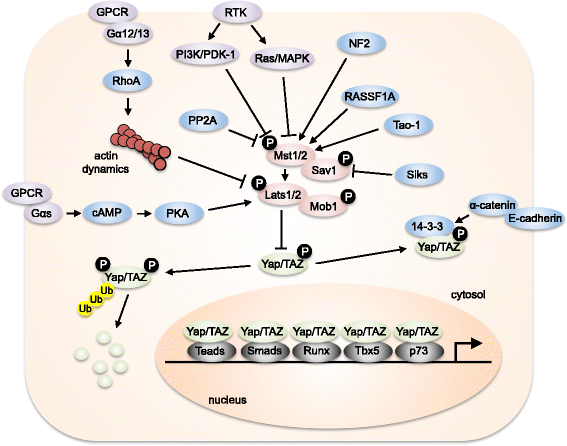

Supplement: Supplementary file 1 — Authors’ original file for figure 1 [file 40169_2014_27_MOESM1_ESM.gif]
